# Burden of Chronic obstructive pulmonary disease in China: an analysis based on the GBD 2021 compared with the United States

**DOI:** 10.1371/journal.pone.0321470

**Published:** 2025-04-17

**Authors:** Rong Li, Yu Li, Chan Xiong, Wei Gao

**Affiliations:** 1 Acupuncture and massage, Chengdu University of Traditional Chinese Medicine, Chengdu, Sichuan, China; 2 Respiratory and critical care Medicine, The Third Affiliated Hospital of Chengdu University of Traditional Chinese Medicine (West), Chengdu, Sichuan, China; Christian Medical College, INDIA

## Abstract

**Background:**

Chronic obstructive pulmonary disease is an important disease affecting physical health worldwide, and the burden of this disease has been growing since 1991 for both China and the US.

**Objective:**

To examine the changes in the burden of COPD in both China and the US between 1990–2021.

**Methods:**

The joinpoint analysis, age-Period-Cohort analysis, decomposition analysis, predictive analysis methods were used to describe prevalence, incidence, death, and disability-adjusted life years for COPD in China and the US.

**Results:**

Compared with China, all four measures of the US COPD burden were higher than they had been in 1990. The burden of COPD increases with age in China. Conversely, in the US, the burden of COPD is getting younger. The epidemiological changes have contributed to an increasing burden of COPD in the US, but have led to a decline in the burden of COPD in China. By 2042, the number of cases in both countries will rise, especially the death rate in the US.

**Conclusion:**

The burden of COPD will not rapidly decline in the short term, both China and the US, as well as the global community, must take this disease seriously.

## Introduction

Chronic obstructive pulmonary disease (COPD), a heterogeneous pulmonary condition characterized by breathlessness, coughing, and expectoration, is caused by persistent airflow obstruction resulting from bronchitis, bronchiolitis, or lung abnormalities [1]. Its morbidity and mortality are on the rise due to a number of reasons, including air pollution caused by industrialization, COVID-19 outbreak, increasing aging, and smoking. Among them, smoking can destroy the lung functional barrier and further aggravate COPD complications. Previous smoking history increases the risk of COPD in both China and the US [2,3]. COPD is characterized by a high prevalence, high rate of disability, and high mortality [4]. Due to industrialization-induced air pollution, the COVID-19 outbreak, and the aging population, its incidence and mortality rates have been on the rise. The prediction that COPD would become the third leading cause of death globally has indeed proven accurate [5]. A study reports that 90% of COPD-related deaths occur in middle- and low-income countries [6]. As the largest developing country, China’s COPD cases account for approximately 25% of the global total, with a COPD mortality rate of around 13.7% among individuals aged 40 and above [7]. And COPD is now the fourth leading cause of death in the US [8]. The US Healthcare Cost Institute forecasts that the medical costs associated with COPD will soar to $60.5 billion in 2029 [9]. For both nations, the disease burden of COPD is significant.

There exist significant disparities between China and the US in various dimensions such as economic development levels, lifestyle habits, and medical management systems. There have already been many analyses assessing the burden of COPD in both China and the US [7,10–12]. However, the databases for these studies are GBD 2019, which has a relative lag in terms of data availability. Secondly, they have not included prevalence, incidence, mortality, and disability-adjusted life years (DALYs) in their analyses, nor have they employed a comprehensive model. As the GBD database information is updated, it necessitates further data mining to gain a clearer understanding of the latest trends in COPD between the two countries. The inadequacy of such comparative analyses may hinder China and the US in identifying their respective shortcomings in public health policies and practices, as well as limit their learning of effective strategies and experiences for COPD. A comparative analysis of the trends and characteristics of COPD burden in both countries can provide directional guidance for future interventions, as well as crucial insights into identifying areas that require improvement and adopting successful practices from other nations.

In summary, this paper aims to use different kinds of model to analysis distinct characteristics of two countries of COPD burden to provide guidance on reducing COPD burden in both China and the US, and focus on different age groups, gender, occupation, rural population, urban population and other factors on the impact of disease burden, thereby fostering the overall health of their respective populations.

## Methods

### Trend analysis

Our study employed the Joinpoint 5.2.0 software developed by the American Cancer Center for segmental local non-linear regression analysis of disease trends between 1990 and 2021 (Download Joinpoint Desktop Software (cancer.gov)). Segmented regression analysis is particularly useful in analyzing trend changes or explaining the variations in the impact of predictors on the response variable. These remarkable shifts are represented by inflection points, which divide the overall trend line into several sub-sections, each indicated by an annual percentage change (APC) that denotes the degree of variation. We also estimated average annual percentage changes (AAPC) from 1990 to 2021 to gain a clearer understanding of trend changes. When APC/AAPC values and their upper 95% CI exceed zero, it indicates that the response variable is trending upwards over a certain period; Conversely, when APC/AAPC values fall below zero, it suggests a descending trend [13].

The Age-period-cohort (APC) model can investigate the impact of these factors on changes in the burden of COPD from three dimensions: age, period, and birth year. APC model can analyze the influence of these factors on COPD burden from three independent time dimensions: age effect, time effect and cohort effect. The general form of its model can be expressed as:


$${\text{Y}}_{\text{ij}}=\mu +\alpha {\text{age}}_{\text{i}}+\beta {\text{period}}_{\text{j}}+\gamma {\text{cohort}}_{\text{k}}+\in_{\text{ij}}$$

represents the response variable, and μ is the population mean. is an age effect (i denotes age group). is the period effect (j denotes period). is the queue effect (k stands for queue). represents random error term.

However, the APC model itself has a collinearity problem, that is, there is a complete linear dependence relationship between the three factors of age, cohort and period (cohort = period − age), so the traditional regression method cannot uniquely estimate the independent effects of age, period and cohort. Therefore, the intrinsic estimator (IE) algorithm is used in this study to eliminate collinearity by limiting parameter space with linear constraints, so as to provide unique, stable and small deviation estimation results [14]. Given the specificity of the IE algorithm, we have reclassified the data into consecutive five-year age groups (15–20, 25–30, etc.). The periods were also divided into consecutive five-year periods (1992–1997, 1997–2002…2017 to 2021), and thus generating five consecutive birth cohorts (1897–1901, 1902–1906…2002 to 2006). Subsequently, the disease burden for specific ages, periods, birth cohorts was represented relative to the average composite level for all ages, periods, and birth cohorts, utilizing the Relative Risk (RR) index and the 95% confidence space(95%CI).

Decomposition analysis is a quantitative method for determining the extent to which differences in a single factor contribute to overall value differences, which helps reveal major heterogeneity in population and epidemiological trends [15]. To discern the factors that shaped the trend changes in COPD burden between 1990 and 2001, we performed a decomposition analysis on age structures, population sizes, and epidemiological shifts, grouping by gender [16]. All figures are done by R 4.3.3 software. The change of age structure is mainly manifested as aging. If the elderly population increases, the decomposition analysis shows the contribution rate of age structure change after controlling the other two factors. Similarly, it can show the contribution of population growth and epidemiological factors such as smoking, air pollution, and policy changes to the burden of COPD.

### Predictive analytics

We employed Bayesian age-period-cohort (BAPC) analysis to predict the COPD burden in China and the US from 2021 to 2046, providing data support for future planning in terms of COPD prevention and treatment. BAPC and INLA (Integrated Nested Laplace Approximation) software packages within R 4.3.3 software for the BAPC model predictions and graphical illustrations.

## Results

### A joinpoint analysis of COPD burden in China and the US between 1990 and 2021

**Fig 1:**
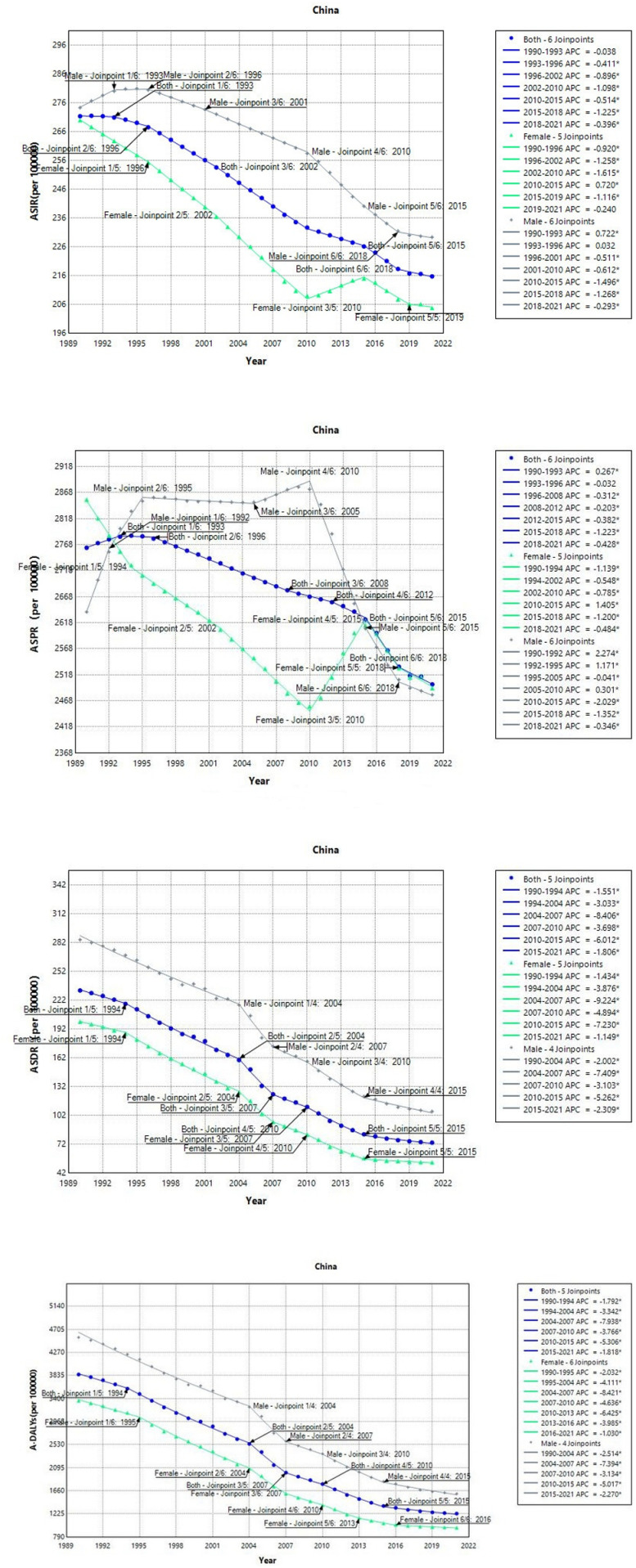
Joinpoint regression analysis of COPD burden in China from 1990 to 2021 (ASIR: age-standardized incidence rate; ASPR: age-standardized prevalence rate; ASDR: age-standardized death rate; A-DALYs: age-standardized disability-adjusted life-years rate).

In China from 1990 to 2021, the age-standardized incidence rate (ASIR), age-standardized prevalence rate (ASPR), age-standardized death rate (ASDR), and age-standardized disability-adjusted life-years rate (A-DALYs) all exhibited a downward trend, particularly for ASDR and A-DALYs, which showed a more significant decrease compared to the previous two periods (Fig 1). The AAPC of ASIR dropped 0.741% (95% CI: -0.741, -0.712) in the period between 1990 and 1996. ASIR was consistently higher in men than in the general population, and lower in women. However, the trend has been on a decline since 2001, particularly between 2010 and 2018, with a faster pace of decrease. Female infertility rates as measured by the ASIR showed a steady decline since 1990 until 2010, after which a third inflection point emerged and a rising trend persisted, with a subsequent decrease observed only after 2015. Men exhibit persistently higher ASIRs compared to the overall population. The ASPR trend as a whole appears to be on a downward trajectory, with an overall decline of 0.322% (95% CI: -0.36, -0.283) over a span of 31 years, with six turning points observed in total since the beginning of 2015. Two major peaks of ASPR were observed among males, one during the years 1990–1995 and another between 2006–2010, which contradicted the overall trend. Not until after 2010 did ASPR begin to decline rapidly, with a total AAPC drop of 0.203% (95% CI: -0.246, -0.159). A spike in the female ASPR occurred during this 31-year period, specifically between 2010 and 2015. Only after 2015 did the trend begin to reverse. Over the years, the ASDR and A-DALYs for COPD have shown a downward trend in both aggregate trends as well as by gender, all having traversed through five critical junctures. The overall ASDR’s AAPC (3.693%, 95% CI: -4.002, -3.382) showed a slightly higher decrease compared to A-DALYs (3.667%, 95% CI: -3.942, -3.409). In men, ASDR and A-DALYs were consistently higher than the overall averages, while in women, they were lower. Gender disparities in changes have shown a consistent downward trend.

**Fig 2:**
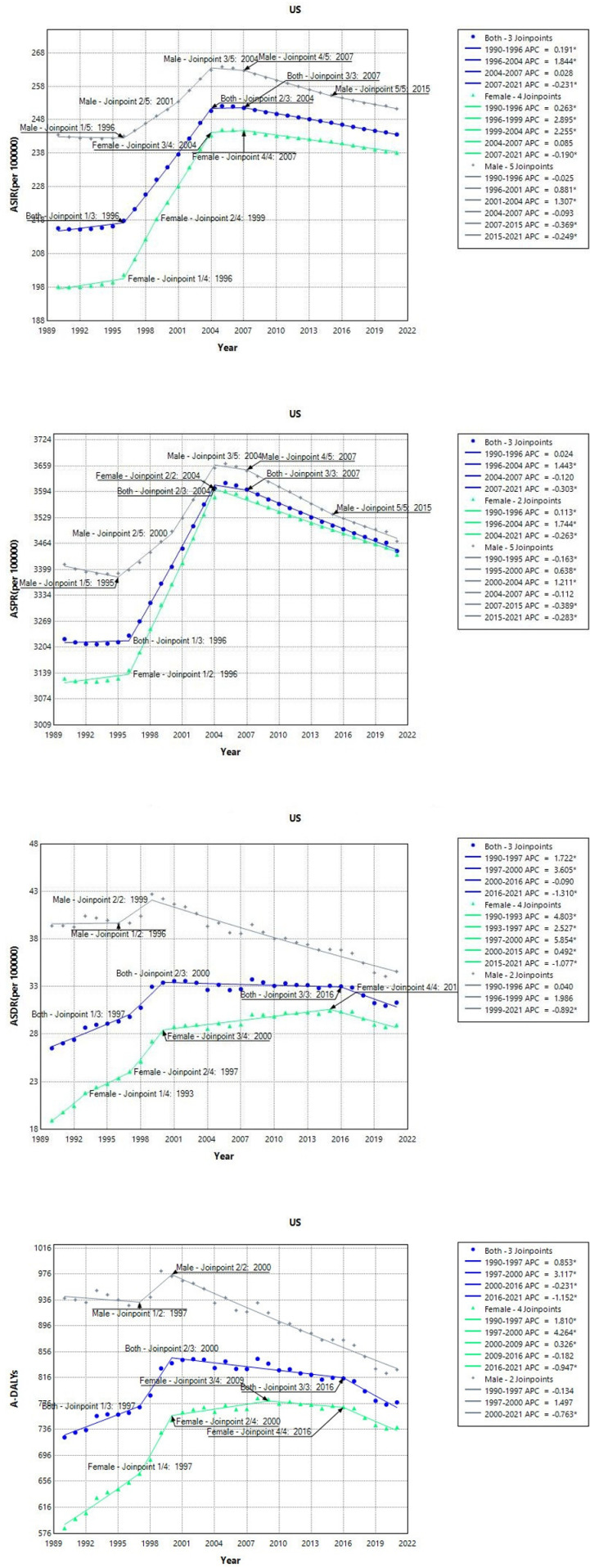
Joinpoint regression analysis of COPD burden in the US from 1990 to 2021 (ASIR: age-standardized incidence rate; ASPR: age-standardized prevalence rate; ASDR: age-standardized death rate; A-DALYs: age-standardized disability-adjusted life-years rate).

Unlike in China, the burden of COPD in the US has consistently risen over the past three decades, particularly between 1996 and 2004 (Fig 2). AAPC rose by 0.407% (95% CI: 0.386, 0.429), with women exhibiting a higher increase compared to the overall population (AAPC: 0.611%, 95% CI: 0.545, 0.677), especially in ASIR and A-DALY. ASPR experienced a general increase across four milestones, with rapid growth from 1996 to 2004, after which it began to decline swiftly. Over the past 31 years, the AAPC rose by 0.226% (95% CI: 0.198, 0.254), which is not a particularly rapid pace compared to China’s growth and decline in ASDR. Male ASDR has experienced three peaks since 1999, with a continuous decline since then. The AAPC decreased by 0.437% (95%CI: -0.762%, -0.11%) over time. But ASDR remains above the average. Similar to ASDR trends, the overall and female A-DALYs exhibited a rapid increase from 1997 to 2000 before stabilizing until gradually declining after 2016. The incidence of A-DALYs among males increased rapidly from 1997 to 2000, before declining sharply afterwards. The AAPC over the entire period showed an overall decrease of 0.405% (95% CI: -0.638%, -0.17%). AAPC value can be seen in Table 1.

**Table 1:** Changes of AAPC values in China and the United States from 1990 to 2021.

Location	Gender	ASIR		ASPR		ASDR		A-DALYs	
China		AAPC	95%CI	AAPC	95%CI	AAPC	95%CI	AAPC	95%CI
	Both	-0.741	(-0.741,-0.712)	-0.322	(-0.36,-0.283)	-3.693	(-4.002,-3.382)	-3.667	(-3.942,-3.409)
	Female	-0.885	(-0.926,-0.843)	-0.431	(-0.478,-0.383)	-4.226	(-4.607,-3.843)	-3.982	(-4.342,-3.621)
	Male	-0.582	(-0.6,-0.563)	-0.203	(-0.246,-0.159)	-3.234	(-3.58,-2.886)	-3.416	(-3.723,-3.109)
US	Both	0.407	(0.386,0.429)	0.226	(0.198,0.254)	0.47	(-0.137,0.804)	0.183	(-0.052,-0.418)
	Female	0.611	(0.545,0.677)	0.324	(0.306,0.342)	1.364	(1.043,1.685)	0.712	(-0.481,-0.942)
	Male	0.11	(0.084,0.136)	0.066	(0.024,0.107)	-0.437	(-0.762,-0.11)	-0.405	(-0.638,-0.17)

AAPC: Average Annual Percentage Change; ASIR: Annualized Standardized Incidence Rate; ASPR: Annualized Standardized prevalence Rate; ASDR: Annualized Standardized Death Rate; A-DALYs: Annualized Standardized DALYs Rate.

### The Age-Period-Cohort (APC) analysis of the burden of COPD in China and the US

**Fig 3:**
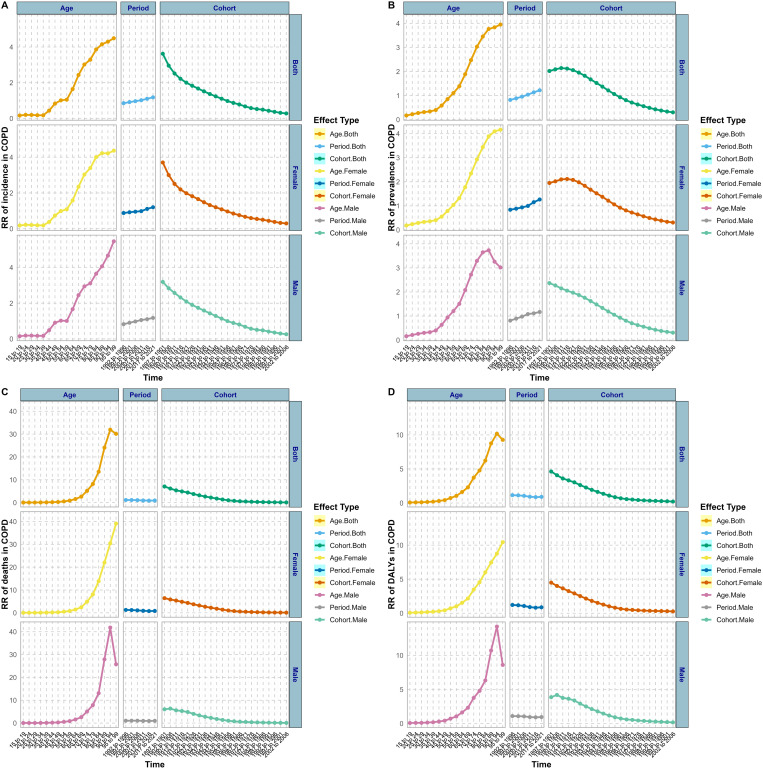
The effects of age, period, and birth cohort on the relative risk of COPD burden in China (RR: relative risk).

Fig 3 illustrates the impact of age-period-cohort factors on the incidence, prevalence, death, and DALYs for COPD in China from 1990 to 2021. Age differences have a relatively greater impact on the disease burden of COPD. The overall and female prevalence, as well as their risk of developing a disease, exhibit an escalating trend. Conversely, men’s risk of contracting COPD decreases after the age of 85–89 (RR_ages 85–89_ = 4.06, 95% CI: 1.39–1.4). Both sexes attained peak incidence rates in the age of 95–99, with an elevated RR for males (RR_aged 95–99 years_ = 5.46, 95% CI: 1.68–1.72) compared to females (RR_aged 95–99 years_ = 4.37, 95% CI: 1.46–1.48). Only female’s RR values of mortality and DALYs continued to rise, while the RR values of all and men decreased after 90–94 years of age, especially the RR values of male mortality decreased by about 62% after 90–94 years of age. The period effect on China’s COPD incidence and prevalence continued to show an upward trend. During the period from 1992 to 2021, the overall incidence and prevalence rates respectively rose by approximately 28% and 33%. Mortality and DALYs have remained relatively stable, with a slight downward trend in DALYs. The cohort effect on the incidence, prevalence, mortality, and DALYs of COPD in China exhibited a stable downward trend, with a smaller burden of COPD occurring among those born later.

**Fig 4:**
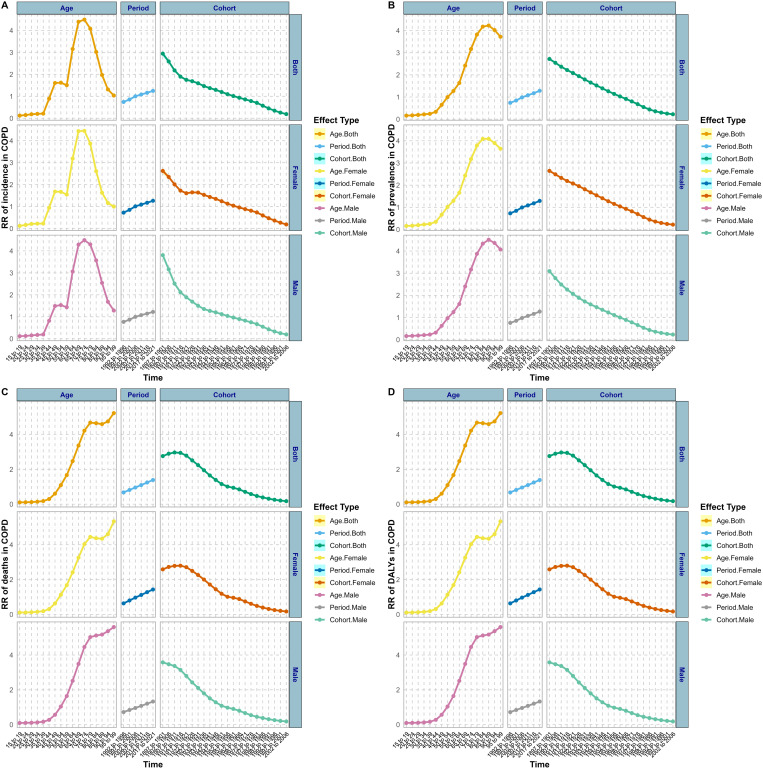
The effects of age, period, and birth cohort on the relative risk of COPD burden in the US (RR: relative risk).

Compared with China’s APC effect, the overall prevalence trend in the US exhibited an initial increase followed by a decrease during the control period and among birth cohorts (Fig 4). The overall incidence rate rose by approximately 86% from ages 35 to 55, and then rapidly increased from 55 to 80 years old, with an RR value increase of about 63%. From the age of eighty, there is a decline trend in place. In terms of the trend influenced by age factors on the prevalence of COPD in the US, it parallels that of China. However, mortality rates increase with age, without experiencing a decline. And in the US, the age effect on DALYs for COPD is growing steadily, with no decline observed among males, which contrasts with China’s pattern. Period effects in the US burden of COPD showed an upward trend. However, the birth cohort effect on US COPD burden shows a declining trend, similar to China. Taking a comprehensive view, the APC effect on male and female COPD burden in the US is relatively minor.

### A decomposition analysis of the burden of COPD in both China and the US

**Fig 5:**
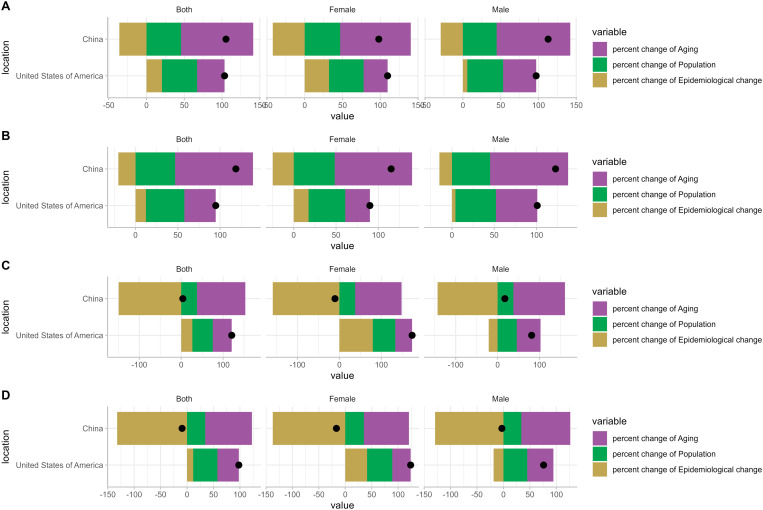
Changes in the burden of COPD by aging, population growth, and epidemiological changes by sex subgroups in China and the US, 1990-2019 (Black dots indicate the overall value of changes resulting from all three components. For each component, the size of the positive value indicates an increase in the corresponding burden of COPD attributed to that component; The size of a negative value indicates a reduction in the corresponding burden of COPD attributed to the component).

The most obvious outcome is that epidemiological changes contribute negatively to China’s COPD burden. Compared to population changes, aging is the primary driver behind the increase in China’s COPD burden. Aging has led to an approximate 115% increase in the mortality rate of COPD in China. Gender groups also exhibit similar changes in trend. Aging, demographic changes, and epidemiological shifts all contribute positively to the burden in the US, particularly in terms of demographic changes. Only epidemiological changes brought about a 21% decrease in the mortality rate among American men. The trend amongst women is similar to that of the overall population. Table 2 to be included in the document. (Fig 5)

**Table 2:** Decomposition analysis of COPD burden in China and the US from 1990 to 2021.

Location	Sex	Measure	Percent change of Aging	Percent change of Population	Percent change of Epidemiological change	Overall percent change
China	Male	Deaths	123.154	37.4927	-143.365	17.28129
		DALYs	92.76143	33.65376	-129.393	-2.97754
		Prevalence	92.071	45.18841	-14.7952	122.4642
		Incidence	97.57482	44.59019	-29.3527	112.8123
	Female	Deaths	110.715	37.73388	-159.061	-10.6124
		DALYs	85.28565	35.11681	-137.149	-16.7467
		Prevalence	91.41992	48.47459	-24.781	115.1136
		Incidence	93.44611	46.92049	-42.3037	98.06292
	Both	Deaths	115.4755	37.5836	-149.223	3.835595
		DALYs	88.31433	34.37366	-132.104	-9.41596
		Prevalence	92.04788	46.80021	-20.2761	118.572
		Incidence	95.4705	45.71877	-35.9844	105.2049
US	Male	Deaths	56.72349	45.65648	-21.2329	81.14711
		DALYs	49.56478	44.88763	-18.602	75.85036
		Prevalence	48.78767	47.93328	4.103325	100.8243
		Incidence	43.76749	47.44295	5.773855	96.98429
	Female	Deaths	39.98688	53.68125	79.78276	173.4509
		DALYs	34.7808	47.55133	41.31236	123.6445
		Prevalence	29.16776	43.31263	17.52192	90.00231
		Incidence	31.6858	45.82546	32.21879	109.7301
	Both	Deaths	45.33674	48.67156	26.56629	120.5746
		DALYs	40.44231	45.87598	11.57717	97.89546
		Prevalence	37.10048	45.42306	12.26288	94.78642
		Incidence	36.42497	46.53845	20.39825	103.3617

### A predictive analysis of the burden of COPD in both China and the US

**Fig 6:**
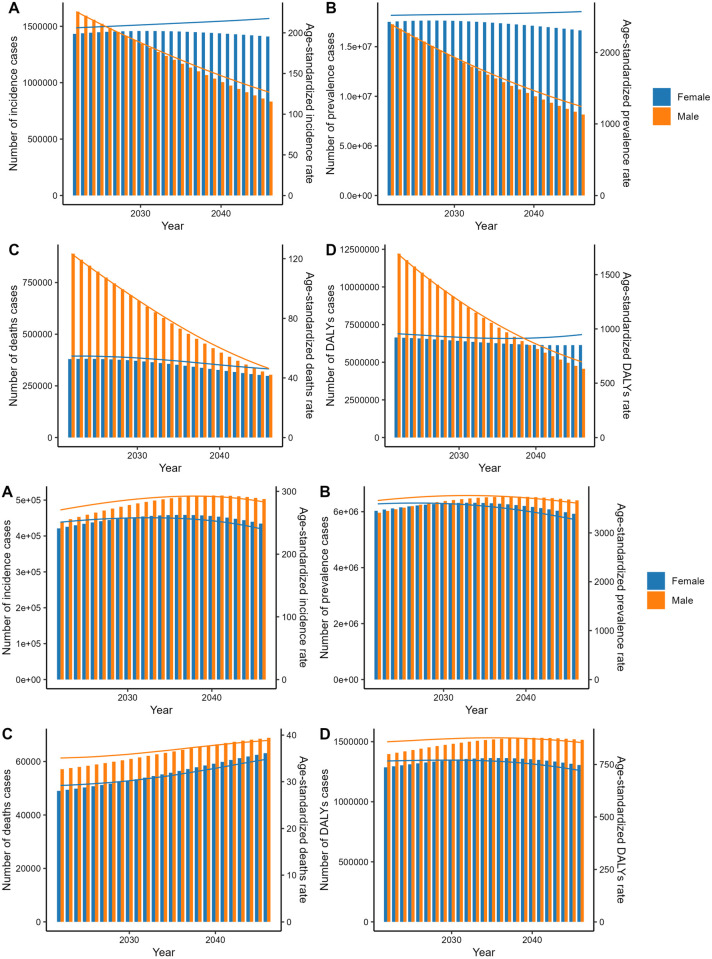
The predicted case number and ASR of the burden of COPD to 2042 in China and the US (ASR: age-standardized rate; Above is China, below is the US).

It can be discerned that the overall COPD burden among Chinese men showed a downward trend from 2021 to 2046, with females’ change more balanced. However, women’s ASIR and ASPR will exceed those of men, with A-DALYs projected to surpass males post-2024. In general, the US forecasts changes in trends with more stability than China, but the caseloads will continue to climb, with significantly higher mortality rates for both men. Specific caseloads and ASR values are as follows. (Table 3, Table 4, Fig 6)

**Table 3:** The predicted case number and ASR of burden of COPD to 2042 in China.

Year	Gender	ASIR	Numer of incidence cases	ASPR	Numer of prevalence cases	ASDR	Numer of deaths cases	A-DALYs	Numer of DALYs cases
2022	Female	206.118	1630064	2517.971	17262470	54.63	889732.6	955.584	12206581
2023		206.55	1594266	2520.364	16824970	54.659	860631.2	951.289	11779699
2024		206.991	1558486	2522.395	16389480	54.65	831871.7	947.276	11362334
2025		207.441	1522616	2524.092	15954720	54.558	803161.3	943.029	10951448
2026		207.874	1486739	2525.744	15520374	54.399	774556.4	938.557	10547414
2027		208.298	1450905	2527.398	15087206	54.201	746154.4	934.1	10151274
2028		208.728	1415145	2528.92	14658080	53.98	717888.4	930.086	9763340
2029		209.179	1379479	2530.256	14234546	53.726	689697.3	926.531	9383235
2030		209.645	1343828	2531.46	13815602	53.406	661546.1	923.149	9010295
2031		210.099	1308207	2532.841	13399963	53.034	633567.1	919.975	8645437
2032		210.541	1272701	2534.418	12988317	52.63	605972.2	917.163	8290404
2033		210.984	1237498	2536.023	12584233	52.214	578958.1	915.037	7947205
2034		211.441	1202710	2537.596	12189518	51.784	552586.4	913.625	7616018
2035		211.909	1168328	2539.204	11803440	51.321	526865.5	912.78	7296172
2036		212.366	1134358	2541.116	11424348	50.834	501879.3	912.427	6987750
2037		212.812	1100887	2543.315	11052600	50.322	477728.2	912.543	6691378
2038		213.264	1068130	2545.621	10691482	49.802	454539.4	913.307	6408289
2039		213.738	1036195	2547.976	10342309	49.277	432351.5	914.796	6138359
2040		214.236	1005015	2550.448	10003686	48.755	411149.3	917.076	5880587
2041		214.739	974510.4	2553.228	9673313	48.249	390910.7	920.117	5634170
2042		215.252	944679.6	2556.289	9350914	47.752	371613.1	923.915	5398920
2043		215.791	915625.5	2559.481	9038868	47.277	353280.2	928.566	5175288
2044		216.369	887376.4	2562.79	8737911	46.831	335904.2	934.138	4962792
2045		216.983	859834.1	2566.271	8446659	46.419	319477.2	940.681	4760529
2046		217.597	832857.7	2569.988	8162726	46.041	303965.3	947.957	4567592
2022	Male	225.998	1432906	2393.332	17504590	123.356	379783.6	1692.365	6643089
2023		220.88	1438360	2331.037	17551171	119.237	380631.2	1632.033	6624534
2024		215.874	1443266	2270.187	17587635	115.227	381050.7	1573.853	6604971
2025		210.971	1447510	2210.657	17612973	111.285	380704.2	1517.412	6580406
2026		206.176	1450909	2152.317	17629055	107.413	379691.5	1462.682	6550888
2027		201.475	1453606	2095.029	17637463	103.612	378242.2	1409.619	6518621
2028		196.863	1455701	2039.112	17637098	99.867	376466	1358.196	6486574
2029		192.336	1457300	1984.678	17627693	96.162	374298.8	1308.275	6454923
2030		187.883	1458310	1931.582	17609034	92.492	371493.2	1259.744	6421496
2031		183.495	1458551	1879.545	17583474	88.867	368169.3	1212.652	6386644
2032		179.165	1458135	1828.439	17552495	85.306	364498	1167.087	6351952
2033		174.908	1457176	1778.66	17515223	81.83	360621.2	1123.261	6319765
2034		170.731	1455780	1730.369	17471470	78.443	356532.8	1081.136	6290348
2035		166.631	1453901	1683.444	17421400	75.143	352109.9	1040.603	6262557
2036		162.598	1451412	1637.562	17367252	71.939	347424.9	1001.621	6235984
2037		158.63	1448443	1592.601	17310313	68.837	342503.5	964.18	6210951
2038		154.747	1445143	1548.943	17249902	65.852	337471.2	928.41	6188846
2039		150.96	1441645	1506.744	17185921	62.988	332370.4	894.281	6170235
2040		147.268	1437907	1465.873	17118089	60.247	327234.2	861.702	6155231
2041		143.657	1433792	1425.986	17047659	57.626	322155.4	830.558	6143531
2042		140.119	1429396	1386.97	16975205	55.119	317100.1	800.792	6135316
2043		136.67	1424804	1349.183	16899534	52.732	312160.1	772.487	6131061
2044		133.318	1420084	1312.769	16820243	50.466	307362	745.602	6130985
2045		130.054	1415137	1277.595	16736924	48.322	302736.4	720.052	6135014
2046		126.857	1409724	1243.31	16649899	46.299	298278.2	695.715	6141426

ASIR: age-standardized incidence rate; ASPR: age-standardized prevalence rate; ASDR: age-standardized death rate; A-DALYs: age-standardized disability-adjusted life-years rate.

**Table 4:** The predicted case number and ASR of burden of COPD to 2042 in the US.

Year	Gender	ASIR	Numer of incidence cases	ASPR	Numer of prevalence cases	ASDR	Numer of deaths cases	A-DALYs	Numer of DALYs cases
2022	Female	250.871	440393.2	3593.187	5964305	29.203	57149.53	766.53	1397082
2023		252.126	446876.5	3599.778	6027518	29.283	57565.85	767.286	1408595
2024		253.268	453162.8	3604.665	6087281	29.375	57987.54	768.081	1419750
2025		254.294	459287.4	3607.942	6144345	29.479	58433	768.874	1430888
2026		255.197	465228.2	3609.76	6199148	29.599	58919.27	769.538	1442227
2027		255.978	470929.5	3610.139	6251070	29.731	59430.84	770.044	1453380
2028		256.627	476356.7	3608.722	6299033	29.878	59949.01	770.351	1463901
2029		257.147	481486.6	3605.448	6342528	30.04	60463.73	770.556	1473613
2030		257.541	486323.3	3600.376	6381885	30.216	60993.11	770.638	1482833
2031		257.797	490841.1	3593.547	6417642	30.418	61557.51	770.46	1491869
2032		257.92	495005.3	3584.949	6449436	30.641	62138.72	769.944	1500343
2033		257.908	498788.1	3574.4	6476383	30.874	62707.08	769.009	1507734
2034		257.762	502182.2	3561.917	6498248	31.116	63255.11	767.785	1513916
2035		257.475	505185.3	3547.55	6515384	31.37	63804.73	766.301	1519284
2036		257.016	507731.6	3531.271	6528110	31.653	64376.04	764.455	1524160
2037		256.371	509790.3	3513.05	6536265	31.96	64950.15	762.131	1528203
2038		255.532	511337.1	3492.902	6539353	32.268	65488.95	759.235	1530912
2039		254.492	512355.4	3470.936	6537222	32.572	65982.66	755.911	1532165
2040		253.231	512836.4	3447.219	6530327	32.878	66455.83	752.241	1532417
2041		251.705	512723.2	3421.734	6519345	33.206	66933.82	748.197	1532105
2042		249.899	511999.5	3394.464	6504329	33.555	67402.4	743.64	1530971
2043		247.815	510659.3	3365.628	6485123	33.893	67823.66	738.49	1528605
2044		245.454	508712.4	3335.393	6461826	34.213	68191.58	732.897	1524955
2045		242.807	506179.3	3303.821	6435070	34.523	68534.91	726.981	1520530
2046		239.848	503016.1	3270.905	6405490	34.846	68879.72	720.794	1515749
2022	Male	270.404	421172.8	3662.121	6032395	35.09	49028.03	857.817	1286883
2023		272.786	425634.6	3679.367	6077072	35.14	49435.48	859.846	1295317
2024		275.061	429866.7	3694.861	6118116	35.197	49857.56	861.761	1303647
2025		277.229	433897.5	3708.77	6156164	35.271	50299.49	863.694	1311915
2026		279.284	437703.9	3721.446	6191322	35.37	50766.31	865.791	1319882
2027		281.229	441229.6	3733.009	6222807	35.491	51246.92	867.928	1327328
2028		283.042	444462.5	3742.763	6250084	35.621	51747.19	869.822	1334200
2029		284.718	447388.9	3750.539	6272813	35.754	52263.36	871.395	1340624
2030		286.257	450018.5	3756.47	6291188	35.901	52799.37	872.817	1346589
2031		287.639	452338.8	3760.822	6305351	36.074	53372.62	874.255	1351873
2032		288.868	454318.5	3763.674	6314772	36.262	53972.93	875.55	1356231
2033		289.931	455948.8	3764.535	6319087	36.45	54581.37	876.402	1359511
2034		290.828	457226.4	3763.324	6318239	36.633	55194.09	876.753	1361921
2035		291.553	458148.3	3760.168	6312463	36.823	55820.02	876.811	1363546
2036		292.072	458653.3	3755.282	6301675	37.032	56485.47	876.77	1364196
2037		292.377	458702.3	3748.701	6285593	37.25	57183.49	876.46	1363614
2038		292.457	458274.8	3740.159	6264220	37.456	57870.09	875.6	1361622
2039		292.313	457347.5	3729.667	6237629	37.645	58534.56	874.143	1358449
2040		291.932	455898.8	3717.381	6206136	37.83	59190.6	872.326	1354283
2041		291.272	453864.4	3703.562	6169937	38.024	59874.97	870.37	1349119
2042		290.325	451218.8	3688.225	6129053	38.22	60586.88	868.124	1342717
2043		289.091	447961.5	3671.309	6083864	38.396	61266.82	865.362	1334929
2044		287.576	444097.7	3652.876	6034707	38.549	61900.89	862.058	1326026
2045		285.775	439640.2	3633.062	5982084	38.693	62509.62	858.449	1316312
2046		283.659	434549.8	3612.16	5926128	38.842	63133.8	854.756	1305912

ASIR: age-standardized incidence rate; ASPR: age-standardized prevalence rate; ASDR: age-standardized death rate; A-DALYs: age-standardized disability-adjusted life-years rate.

## Discussion

This paper is based on GBD 2021 high-end database, examining the COPD burden of the specific changes from 1990 to 2021. The changes in COPD burden in both countries from 2022 to 2046 were predicted. In 2021, we observed that in the US, COPD burden increased compared to 1990, while China experienced a sustained decrease. Despite the fact that the incidence and prevalence of COPD in the US were higher than those in China in 2021. China had a higher mortality rate and DALYS for COPD compared to the US. By 2046, the overall burden of COPD in China for males is expected to gradually decline, with minimal changes in the disease burden in the US; however, the mortality rate is predicted to show a mild increase.

The incidence and prevalence in the US have experienced overall growth trend, ultimately remaining higher than that of China. As evidenced by trends and decomposition analyses, ASIR and ASPR in the US saw rapid increases in 1996 but then declined swiftly in 2004. The most significant impact is demographic changes. A salient feature of the US is its racial diversity, with Latinx, Hispanic, and African Americans having a higher likelihood of developing COPD, largely due to environmental factors and unequal access to healthcare [17]. And yet, one must account for genetic defects that differentiate populations, such as the increased risk of α-1 antitrypsin deficiency in African Americans for developing COPD [18]. We infer the rise in prevalence and incidence in 1996 was due to changes in the racial composition of the US population and total population growth. Of course, demographic aging and epidemiological changes also contributed positively to its growth [19]. Following the US industrial research and development renaissance of 1996, there was a further 15% increase in occupational exposure risks. In 2004, the American economy had entered a mature phase of full-fledged growth. President George W. Bush proposed to establish a comprehensive health-care network to facilitate the sharing of health information across institutions, promoting resource sharing among medical facilities nationwide. This not only enhanced medical standards but also fostered the proliferation of multiple medical establishments, which undoubtedly reducing the prevalence of COPD. However, the COPD prevalence and incidence in the US remain higher than those in China, an observation that contrasts with Perret JL’s finding that COPD prevalence is relatively high in middle-income and low-income SDI countries [20]. Due to different national conditions, there are differences in the healthcare systems between the two countries. As of 2023, China’s medical insurance coverage rate has reached 95%, achieving “universal medical insurance” [21]. This will increase the enthusiasm of COPD patients to seek medical treatment and thereby reduce the incidence of the disease. In contrast, the healthcare system in the US adopts a mixed public-private model, and the probability of rehabilitation for those with public insurance and the uninsured will be lower.The reasons for this lie predominantly in changes to population size. Notwithstanding this, one must also consider that the US has a more comprehensive healthcare system, which leads to better documentation of COPD cases. Moreover, diagnostic criteria for COPD in China and the US may differ due to regional peculiarities. The findings of this study also suggest that the US must re-examine its efforts to reduce the prevalence and incidence of COPD.

The US COPD ASIR stands out in its specificity compared with that in China. In the US, the age group most affected by COPD is 55–80 years old, while in China, it is those aged 79 and above. The incidence of COPD in the US is showing a trend of younger onset. In recent years, the usage rate of e-cigarettes among young people in the US has been relatively high. By 2020, the rate of e-cigarette use among teenagers had reached 19.2%. E-cigarettes can exacerbate cardiopulmonary toxicity and increase the risk and incidence of COPD in adulthood. Of course, the impact of an aging population and epidemiological changes cannot be ignored [22]. Another prominent feature is that the ASIR of American women increased more than that of men from the mid-20th century to the early 21st century. This was because tobacco companies heavily promoted cigarettes to women in the mid-20th century, and cigarettes were regarded as a symbol of women’s liberation. This led to an increase in the smoking rate among women. Secondly, due to the inherent social concept of “men are superior to women”, women tended to conceal their smoking history and missed the opportunity for medical consultation. Low-income women had more opportunities for occupational exposure. Low-quality women delayed seeking medical treatment until they developed symptoms of COPD, which were all reasons for the increase in the incidence of COPD among women [23].

Despite China’s overall decline in COPD prevalence and incidence, gender disparities exhibit a peculiar pattern in terms of disease rates. The disparity in prevalence between men and women has been substantial, with this difference only gradually reducing post-2015. In the male population, the incidence rates plummeted post-2010, while in females, it rose sharply post-2010 before declining gradually until after 2015. Moreover, the incidence among men appears to have fallen since 2015. Of these factors, population size changes made the greatest contribution, which we analyzed was due to the severe gender imbalance in China, with more men than women but an increasing number of those affected [24], causing the female prevalence and incidence rates to surpass those of males. Of course, demographic shifts and epidemiological changes have significant implications. Pathological changes contribute negatively. We speculate that since the implementation of gender employment equality policies in 2010, the employment rates among middle-aged and young women have increased, thereby heightening their occupational exposure risks. Subsequent policies adopted in 2012 and 2019 to protect women’s employment, among others, led to another decline in the incidence rate [25]. Certainly, female exposure to secondhand smoke and kitchen pollutants cannot be ruled out, but males have seen a lower prevalence rate post-2015, speculated to be linked to the nation’s efforts in treating COPD among males, with further investigation required. This also suggests that China must address disparities in COPD incidence between men and women. Following China’s implementation of its National Plan for Chronic Disease Control and Prevention in 2015 [26], the ASPR for COPD has seen a rapid decline. For instance, establishing an integrated cross-departmental coordination mechanism, specifying the division of responsibilities among grassroots health institutions, realizing resource sharing, and implementing tiered, targeted interventions in line with a three-tiered prevention strategy. A range of measures were employed to decrease the prevalence of COPD. Secondly, in 2015, the “most stringent” Environmental Protection Law was enacted, which rigorously tackled environmental issues and reduced the threat to public health posed by pollution.

In 2021, the burden of COPD data between China and the US showed that the majority of COPD deaths occurred among individuals aged 60+ years. As the age advances in China, the ASDR for COPD rises rapidly after 59 years, with an estimated 97% increase by the time one reaches 99. The results revealed that the mortality rate of COPD was positively proportional to age. The decomposition analysis has proven that aging is the most crucial reason for the increasing burden of COPD in China. All this underscores the direct impact of population aging on COPD mortality. China has entered a deeply aging society [27], with 21% of its total population aged 60 or above, a growing labor force, and declining fertility rates. While America’s population of those aged 60 and above constitutes only 16% of its total [28], this figure is significantly lower than that in China. There are more basic diseases in the elderly, the course of disease is long, and the use of multiple antibiotics during hospital admission causes multiple drug resistance. As one ages, their muscle composition decreases, leading to sarcopenia in many older individuals, which exacerbates the severity of COPD [29]. All these factors underscore the need to focus on the senior population as a means to reduce mortality rates.

COPD can be diagnosed and treated in its early stages to prevent later exacerbations [30]. According to studies, China’s actual diagnosis rate for COPD is 26.8%, significantly lower than the US’ 68.3% [31]. The specific procedure for diagnosing COPD in China is by no means comprehensive. This can be attributed to partial inadequacy of medical facilities in primary healthcare institutions, insufficient proficiency of medical personnel, unequal distribution of healthcare resources, and an imbalance between population size and available medical resources. This is in contrast to the comprehensive COPD diagnostic, therapeutic, and prognostic systems in place in the US [32]. At present, the revision of GOLD 2023 upgrades the COPD screening assessment tool with the addition of diagnostic measures such as chest CT scans [33]. These measures contribute to an earlier diagnosis of COPD, thereby reducing mortality rates. But achieving satisfactory results will take much longer [34]. Given China’s slower economic development compared to the US, residents’ base salaries cannot meet individual needs, thus impacting the early detection rate of COPD. Early undertreatment of COPD can lead to irreversible lung function damage, thereby increasing the risk of death [35]. The US has a higher level of public awareness regarding COPD healthcare, with residents more readily recognizing the importance of early treatment. And while the US has built out its healthcare system to ensure lost workdays and sleep hours are accounted for, residents have a much more secure foundation upon which to seek medical attention [36]. A series of measures adopted by the US could offer guidance for China in reducing COPD mortality rates.

Subgroup analyses revealed that the male mortality rates were consistently higher in both China and the US. This is closely tied to male lifestyle and occupational environments. Tobacco smoke is known to be a perilous factor contributing to COPD, with males smoking at a rate 31% higher than females [37]. According to GBD 2021, China’s male population had a 44% higher mortality rate due to COPD caused by tobacco smoke inhalation compared to females [38]. According to research, one of the reasons for the growing COPD mortality rate in the US is the increase in smoking rates. In order to reduce mortality, smoking must be controlled. Professional exposure factors are the leading cause of global COPD mortality [39]. Especially in male-dominated work environments, COPD is more prevalent than in female-dominated ones, with a 34% increased risk of mortality due to underlying respiratory system diseases caused by occupational environments. In the US, the professional exposure risk contributes significantly less to COPD mortality than in China, approximately 58%. This can be attributed to differences in the industrialization processes and sector types between China and the US. Professional exposure to COPD primarily occurs during work when inhaling dust, smoke, or toxic gases. This is often observed in industrial settings. China was lagging behind advanced economies like the US in terms of technology and economic development during the first three industrial revolutions, which led to an industry dominated by agriculture and manufacturing. Consequently, an abundance of cheap labor engaged in factory occupations has led to respiratory diseases. In the Fourth Industrial Revolution, China has emerged as a leader in artificial intelligence and chip technology innovations, reducing occupational exposure risks through the conversion of industries, and, of course, more significantly, applying technological innovations to healthcare to enhance medical standards and reduce mortality rates [40].

While China’s A-DALYs show a downward trend, they remain higher than those in the US. For China, the threat to A-DALYs growth for COPD is environmental pollution. The rapid industrialization has brought about negative impacts in the form of pollution, yet China is actively tackling this issue with multiple measures, resulting in significant progress as witnessed by the sustained decrease in A-DALYs. Population aging contributes to an approximately 88% increase in A-DALYs, thus highlighting the need to address the threat posed by aging to DALYs. While the US’s A-DALYs have consistently lagged behind China, their trend has not been one of constant decline. There is a marked change, manifested by a rapid decline in A-DALYs among males since 2000. Decomposition analysis suggests that the negative contribution of epidemiological changes to A-DALYs for males is approximately 18%. Therefore, it is inferred that this improvement in male smoking prevalence is closely linked with the series of anti-smoking policies enacted in the US around the year 2000. China should further learn from America’s tobacco-control policies, as a comprehensive smoke-free policy could reduce COPD mortality [41]. Various measures combining traditional Chinese and Western medicine treatments are adopted to assist in quitting smoking. But the US’s COPD burden is not showing a sustained downward trend, which suggests that more efforts are needed to change the status quo in the country.

For the prediction of future burden of COPD, for the US, the key point is the increase in mortality rate. To control the mortality rate, early prevention of COPD can be carried out. Not only should health education be provided to the public, but pulmonary function tests should also be included in the essential items of physical examinations. The medical insurance system can learn from China and expand the coverage of medical insurance and improve the visit rate. Big data models can be used to monitor the condition of COPD patients. For China, the disease burden of Chinese men will show a downward trend, but it is not obvious for women. This suggests that China should pay more attention to women. The main influencing factors for Chinese women to suffer from COPD are air pollution, second-hand smoke, and occupational exposure. Promote the use of clean fuels, educate women to ventilate more when cooking to reduce the impact of air pollution. Reduce exposure to second-hand smoke through smoking policies, enhance self-protection awareness for women in high-risk occupations, and reduce exposure to harmful gases and dust.

## Conclusion

In conclusion, COPD burden is severe in both countries and worldwide, yet it will not witness significant changes in the next two decades. China should study the measures employed by the US in controlling mortality and DALYs, while the US should learn from China’s approach to reducing prevalence and incidence rates. Countries should work together to reduce the burden of COPD.
